# Incidence and risk factor prevalence of community-acquired pneumonia in adults in primary care in Spain (NEUMO-ES-RISK project)

**DOI:** 10.1186/s12879-016-1974-4

**Published:** 2016-11-07

**Authors:** I. Rivero-Calle, J. Pardo-Seco, P. Aldaz, D. A. Vargas, E. Mascarós, E. Redondo, J. L. Díaz-Maroto, M. Linares-Rufo, M. J. Fierro-Alacio, A. Gil, J. Molina, D. Ocaña, Federico Martinón-Torres, on behalf of NEUMOEXPERTOS group, F. Martinón-Torres, D. Vargas, E. Mascarós, E. Redondo, J. L. Díaz- Maroto, M. Linares-Rufo, A. Gil, J. Molina, D. Ocaña, I. Rivero-Calle

**Affiliations:** 1 Translational Pediatrics and Infectious Diseases Section, Pediatrics Department, Hospital Clínico Universitario de Santiago de Compostela, Travesía da Choupana, s/n, 15706 Santiago de Compostela, Spain; 2 Genetics, Vaccines, Infections and Pediatrics Research Group (GENVIP), Healthcare Research Institute of Santiago de Compostela, Santiago de Compostela, Spain; 3 Member of the Infectious Diseases Prevention Group PAPPS-SEMFYC, Primary Health Care Center San Juan, Pamplona, Spain; 4 Versatile Hospitalization Unit, Hospital de Alta Resolución El Toyo, Agencia Pública Sanitaria, Hospital de Poniente, Almería, Spain; 5 Health Department, Hospital Dr Peset, Primary Care Center Fuente de San Luís, Valencia, Spain; 6 Preventive and Public Health Activities Group SEMERGEN, International Heath Center, Madrid, Spain; 7 Primary Care Health Center Guadalajara, Infectious Diseases Group SEMERGEN, Guadalajara, Spain; 8 Primary Care and Clinical Microbiology, Infectious Diseases Group SEMERGEN, Fundación io, Spain; 9 Primary Care Health Center El Olivillo, Cádiz, Spain; 10 Preventive Medicine and Public Health, Rey Juan Carlos University, Madrid, Spain; 11 Primary Care Respiratory Group, Health Care Center Francia, Fuenlabrada, Madrid Spain; 12 Primary Care Respiratory Group, Health Care Center Algeciras, Algeciras, Spain

**Keywords:** Community-acquired pneumonia, Vaccine-preventable diseases, Risk factors, Incidence, Antibiotics, Primary care

## Abstract

**Background:**

Community-acquired pneumonia (CAP) is a major cause of morbidity and mortality in adults even in developed countries. Several lifestyle factors and comorbidities have been linked to an increased risk, although their prevalence has not been well documented in the primary care setting. The aim of this study is to assess the incidence, risk factor and comorbid conditions distribution of CAP in adults in primary care in Spain.

**Methods:**

Retrospective observational study in adults (>18 years-old) with CAP diagnosed and attended at primary care in Spain between 2009 and 2013, using the Computerized Database for Pharmacoepidemiological Studies in Primary Care (BIFAP).

**Results:**

Twenty-eight thousand four hundred thirteen patient records were retrieved and analyzed. Mean age (standard deviation): 60.5 (20.3) years, 51.7 % males. Global incidence of CAP in adults was estimated at 4.63 per 1000 persons/year. CAP incidence increased progressively with age, ranging from a 1.98 at 18–20 years of age to 23.74 in patients over 90 years of age. According to sex, global CAP incidence was slightly higher in males (5.04) than females (4.26); CAP incidence from 18 to 65 year-olds up was comparable between males (range: 2.18–5.75) and females (range: 1.47–5.21), whereas from 65 years of age, CAP incidence was noticeable higher in males (range: 7.06–36.93) than in females (range: 5.43–19.62). Average prevalence of risk factors was 71.3 %, which increased with age, doubling the risk in males by the age of 75 (females 20 % vs males 40 %). From 55 years of age, at least one risk factor was identified in 85.7 % of cases: one risk factor (23.8 %), two risk factors (23.4 %), three or more risk factors (38.5 %). Major risk factors were: metabolic disease (27.4 %), cardiovascular disease (17.8 %) and diabetes (15.5 %).

**Conclusions:**

The annual incidence of CAP in primary care adults in Spain is high, comparable between males and females up to 65 years of age, but clearly increasing in males from that age. CAP risk increases with age and doubles in males older than 75 years. The majority of CAP cases in patients over 55 years of age is associated to at least one risk factor. The main risk factors associated were metabolic disease, cardiovascular disease, and diabetes.

**Electronic supplementary material:**

The online version of this article (doi:10.1186/s12879-016-1974-4) contains supplementary material, which is available to authorized users.

## Background

Community-acquired pneumonia (CAP) is a major cause of morbidity and mortality in adults even in developed countries [1–3]. CAP leads to high rates of hospitalization, particularly in the elderly, and belongs to the top 5 causes of death globally [4]. Depending on the geographic area analyzed, the estimated annual incidence ranges between 1.6 and 12 cases per 1000 population [5–9].

Several lifestyle factors have been associated with an increased risk of CAP including smoking, alcohol abuse, being underweight, having regular contact with children, and poor dental hygiene, among others [10–12]. Also the presence of comorbid conditions, including chronic respiratory and cardiovascular diseases, cerebrovascular disease, Parkinson’s disease, epilepsy, dementia, dysphagia, HIV or chronic renal or liver disease have been linked to an increased risk of CAP [10–12].

The burden of pneumonia is of considerable importance. However, the prevalence of different lifestyle risk factors and comorbidities in patients seeking medical assistance at primary care level because of pneumonia has not been well documented. This study aims to describe the prevalence of different lifestyle risk factors and comorbidities in patients with CAP at primary care in Spain during a 5 year period (2009–2013).

## Methods

An observational retrospective study was designed to evaluate the incidence of CAP and the prevalence and distribution of risk factors and comorbidities in adults in primary care in Spain, attended between 2009 and 2013.

Data collection was performed using the Computerized Database for Pharmacoepidemiological Studies in Primary Care (BIFAP). BIFAP is a computerized database aggregating information provided by 2692 general practitioners and pediatricians working in the Spanish National Health Service. The ownership of this database is public, and its sole purpose is to enable epidemiological studies, and in particular pharmacoepidemiological studies. The information related to these patients is kept completely confidential and strictly compliant with current Spanish and European legislation. Collaboration by general practitioners in BIFAP is personal, individual and voluntary. BIFAP is sponsored by three leading Spanish medical societies in primary care: Sociedad Española de Medicina Familiar y Comunitaria (SEMFYC), Sociedad Española de Médicos de Atención Primaria (SEMERGEN) and Asociación Española de Pediatría de Atención Primaria (AEPAP). The BIFAP database includes clinical and prescription data for around 4.8 million patients.

Eligible study participants were Spanish adults 18 years of age or older, of both sexes, diagnosed of the first episode of CAP between January 2009 and December 2013. Patients had to have been recorded with the doctor for 1 year at least. Patients with previous diagnoses of pneumonia or nosocomial pneumonia were excluded.

All patients were identified when a first register of pneumonia during the study period was detected in their medical history. Patients with a potential diagnosis of pneumonia were selected through a computerized algorithm that identified them through process codes (CIAP R81), or through the occurrence of the word “pneumonia” in the free text history. Cases were included when there was a radiological diagnosis and/or where the diagnosis was reported by another professional in a medical record. Doubtful cases, which involved presumptive diagnosis not confirmed by additional tests, or those where information in the clinical history was insufficient, were excluded.

Different lifestyle risk factors and comorbidities associated to the first register of pneumonia were also retrieved and analyzed (Additional file 1). Comorbidities in BIFAP database are codified according to the Spanish version of the International Classification of Diseases, 9th Revision, Clinical Modification (Modificación Clínica Clasificación Internacional de Enfermedades; CIE-9-MC) and/or the Spanish version of the *International Classification of Primary Care* (CIAP-2/*ICPC-2 PLUS*) (Additional file 1).

## Statistical analysis

Data were presented as mean (standard deviation) or frequency (percentage) as appropriate. We carried out a descriptive analysis of the first episodes of CAP, prevalence of the different lifestyle risk factors and comorbidities stratified by age and gender. The relationship was tested using a binary logistic model. We performed all the analyses using R Software, Version 3.0.2.

## Results

A total of 2,332,622 adult patients (18 years of age or older) were included in the study cohort. 28,413 subjects with a mean age (standard deviation) of 60.5 (20.3) years had a first episode of pneumonia during the study period (Table 1).

**Table 1:** Summary of main characteristics of the study cohort. Data are expressed as % (n)

	NEUMO-ES-RISK total cohort^a^ *N* = 28,413	AGE (years)					
		18–25 years (*N* = 1038)	25–40 years (*N* = 4861)	40–55 years (*N* = 5557)	55–70 years (*N* = 6057)	70–85 years (*N* = 7484)	>85 years (*N* = 3416)
Characteristics of the cohort							
Pneumonia Type							
Pneumonia (unspecified)	57.9 % (16.462)	53.7 % (557)	57.6 % (2.799)	59.3 % (3.297)	60.2 % (3.645)	56.9 % (4.262)	55.7 % (1.902)
Bacterial pneumonia	27.6 % (7.849)	21.2 % (220)	25.9 % (1.261)	26.7 % (1.486)	27.5 % (1.664)	29.7 % (2.226)	29.0 % (992)
Atypical pneumonia	3.5 % (999)	4.4 % (46)	5.2 % (251)	4.8 % (267)	3.7 % (222)	2.2 % (164)	1.4 % (49)
Viral pneumonia	2.8 % (801)	3.4 % (35)	3.8 % (183)	3.9 % (216)	2.8 % (171)	1.9 % (140)	1.6 % (56)
Complicated pneumonia	6.1 % (1.727)	17.1 % (177)	7.1 % (345)	4.7 % (263)	5.2 % (312)	6.1 % (456)	5.1 % (174)
Aspirative pneumonia	1.9 % (551)	0.3 % (3)	0.4 % (20)	0.5 % (26)	0.6 % (39)	3.0 % (224)	7.0 % (239)
Nosocomial pneumonia	0.1 % (24)	0.0 % (0)	0.0 % (2)	0.0 % (2)	0.1 % (4)	0.2 % (12)	0.1 % (4)
Gender							
Female	48.3 % (13.716)	41.4 % (430)	49.6 % (2.411)	50.2 % (2.790)	48.7 % (2.949)	42.8 % (3.203)	56.6 % (1.933)
Male	51.7 % (14.697)	58.6 % (608)	50.4 % (2.450)	49.8 % (2.767)	51.3 % (3.108)	57.2 % (4.281)	43.4 % (1.483)
Risk factors							
Alcoholism	2.5 % (700)	0.0 % (0)	0.8 % (39)	3.6 % (199)	4.2 % (256)	2.5 % (185)	0.6 % (21)
Tobacco	13.0 % (3.702)	8.0 % (83)	13.5 % (656)	20.9 % (1.163)	18.3 % (1.106)	8.3 % (621)	2.1 % (73)
Anemia	12.3 % (3.501)	5.7 % (59)	7.1 % (346)	9.0 % (498)	7.7 % (469)	16.7 % (1.253)	25.6 % (876)
Asthma	6.5 % (1.833)	7.8 % (81)	4.9 % (237)	5.0 % (277)	6.1 % (367)	8.2 % (615)	7.5 % (256)
Bronchitis	2.1 % (591)	0.2 % (2)	0.4 % (20)	0.9 % (51)	2.3 % (138)	3.5 % (265)	3.4 % (115)
COPD	10.8 % (3.055)	0.2 % (2)	0.2 % (9)	3.1 % (173)	11.7 % (709)	21.5 % (1.612)	16.1 % (550)
Low weight	2.9 % (828)	3.6 % (37)	2.5 % (121)	2.4 % (136)	2.4 % (148)	3.6 % (272)	3.3 % (114)
Cardiovascular disease	17.8 % (5.071)	0.5 % (5)	1.7 % (82)	4.3 % (238)	15.5 % (936)	33.0 % (2.469)	39.3 % (1.341)
Cerebrovascular disease	7.8 % (2.221)	0.1 % (1)	0.2 % (12)	0.9 % (48)	4.6 % (281)	14.4 % (1.077)	23.5 % (802)
Bipolar syndrome	0.2 % (63)	0.1 % (1)	0.2 % (8)	0.3 % (17)	0.3 % (16)	0.2 % (17)	0.1 % (4)
Dementia	4.3 % (1.233)	0.0 % (0)	0.0 % (1)	0.0 % (2)	0.9 % (54)	7.2 % (539)	18.6 % (637)
Psychiatric syndrome	0.2 % (71)	0.3 % (3)	0.1 % (4)	0.3 % (15)	0.3 % (16)	0.2 % (15)	0.5 % (18)
Depression	14.8 % (4.197)	2.1 % (22)	6.6 % (323)	12.0 % (667)	17.0 % (1.030)	19.4 % (1.452)	20.6 % (703)
Diabetes	15.5 % (4.415)	0.8 % (8)	1.5 % (75)	5.7 % (314)	17.9 % (1.082)	28.9 % (2.165)	22.6 % (771)
Metabolic disease	27.4 % (7.780)	1.7 % (18)	5.9 % (287)	17.7 % (986)	40.4 % (2.445)	41.5 % (3.106)	27.5 % (938)
Dysphagia	1.5 % (425)	0.4 % (4)	0.6 % (30)	0.6 % (36)	1.5 % (88)	2.0 % (153)	3.3 % (114)
Neurologic disease	1.3 % (362)	0.5 % (5)	0.5 % (22)	0.9 % (52)	1.7 % (100)	1.6 % (117)	1.9 % (66)
Parkinson	1.6 % (447)	0.0 % (0)	0.0 % (0)	0.1 % (3)	0.5 % (33)	3.4 % (258)	4.5 % (153)
Epilepsy	1.9 % (535)	2.0 % (21)	1.6 % (80)	1.7 % (93)	1.9 % (118)	2.0 % (149)	2.2 % (74)
Hepatic disease	1.7 % (472)	0.2 % (2)	0.5 % (23)	1.9 % (107)	2.7 % (165)	2.1 % (155)	0.6 % (20)
Chronic renal disease	0.2 % (44)	0.0 % (0)	0.0 % (0)	0.0 % (1)	0.1 % (6)	0.3 % (21)	0.5 % (16)
Rheumatologic disease	4.7 % (1.335)	0.6 % (6)	0.7 % (35)	1.9 % (104)	4.3 % (261)	7.8 % (582)	10.2 % (347)
HIV	1.0 % (295)	0.2 % (2)	1.4 % (70)	3.5 % (196)	0.4 % (23)	0.1 % (4)	0.0 % (0)
Poor dental hygiene	11.8 % (3.359)	9.1 % (94)	10.0 % (485)	11.5 % (639)	14.3 % (865)	13.6 % (1.021)	7.5 % (255)
Low social status	1.5 % (426)	0.8 % (8)	0.9 % (42)	1.1 % (63)	0.9 % (52)	2.0 % (147)	3.3 % (114)
Pneumococcal vaccination	0.1 % (18)	0.4 % (4)	0.1 % (5)	0.1 % (3)	0.0 % (2)	0.0 % (3)	0.0 % (1)

^a^ Frequencies for categorical variables, number of patients between parenthesis

### Incidence of CAP

Global incidence of CAP in adults was estimated at 4.63 per 1000 persons/year (Table 2). CAP incidence increased progressively with age, ranging from a 1.98 at 18–20 years of age to 23.74 in patients over 90 years of age (Table 2). According to gender, global CAP incidence was slightly higher in males (5.04) than females (4.26) (Table 2). CAP incidence from 18 to 65 year-olds up was comparable between males (range: 2.18–5.75) and females (range: 1.47–5.21), whereas from 65 years of age, CAP incidence was noticeable higher in males (range: 7.06–36.93) than in females (range: 5.43–19.62) (Table 2).

**Table 2:** CAP incidence according to age and gender. Rates expressed per 1000 person-years

	Male				Female				Total			
	Cases	n	p-y	Rate	Cases	n	p-y	Rate	Cases	n	p-y	Rate
18–20 years	175	61,333	70,412.87	2.49	101	61,303	68,905.12	1.47	276	122,636	139,317.98	1.98
20–25 years	433	79,410	196,125.36	2.21	329	89,087	204,046.95	1.61	762	168,497	400,172.31	1.90
25–30 years	566	111,187	260,050.38	2.18	458	122,885	275,279.73	1.66	1024	234,072	535,330.11	1.91
30–35 years	818	134,560	332,654.60	2.46	796	140,318	343,610.41	2.32	1614	274,878	676,265.01	2.39
35–40 years	1070	123,355	336,083.56	3.18	1157	125,302	341,845.69	3.38	2227	248,657	677,929.25	3.29
40–45 years	1074	108,836	306,130.49	3.51	1060	110,765	309,832.41	3.42	2134	219,601	615,962.90	3.46
45–50 years	823	93,700	272,812.94	3.02	833	98,233	280,717.22	2.97	1656	191,933	553,530.15	2.99
50–55 years	874	81,114	239,243.53	3.65	903	88,311	256,471.30	3.52	1777	169,425	495,714.83	3.58
55–60 years	917	71,745	207,713.86	4.41	937	77,261	222,700.73	4.21	1854	149,006	430,414.59	4.31
60–65 years	1097	64,835	190,729.33	5.75	1080	71,241	207,353.03	5.21	2177	136,076	398,082.36	5.47
65–70 years	1098	48,791	155,627.10	7.06	935	54,759	172,088.42	5.43	2033	103,550	327,715.52	6.20
70–75 years	1214	41,283	116,993.45	10.38	890	51,806	143,029.88	6.22	2104	93,089	260,023.33	8.09
75–80 years	1604	36,855	109,640.33	14.63	1115	52,422	149,267.10	7.47	2719	89,277	258,907.43	10.50
80–85 years	1472	25,189	75,766.96	19.43	1204	43,904	125,031.08	9.63	2676	69,093	200,798.03	13.33
85–90 years	978	12,965	39,021.71	25.06	1081	28,978	83,030.03	13.02	2059	41,943	122,051.74	16.87
90–110 years	514	4952	13,917.42	36.93	873	15,937	44,495.90	19.62	1387	20,889	58,413.33	23.74
Total	14,727	1,100,110	2,922,923.88	5.04	13.752	1,232,512	3,227,704.99	4.26	28,479	2,332,622	6,150,628.88	4.63

### Risk factors for CAP

**Fig. 1:**
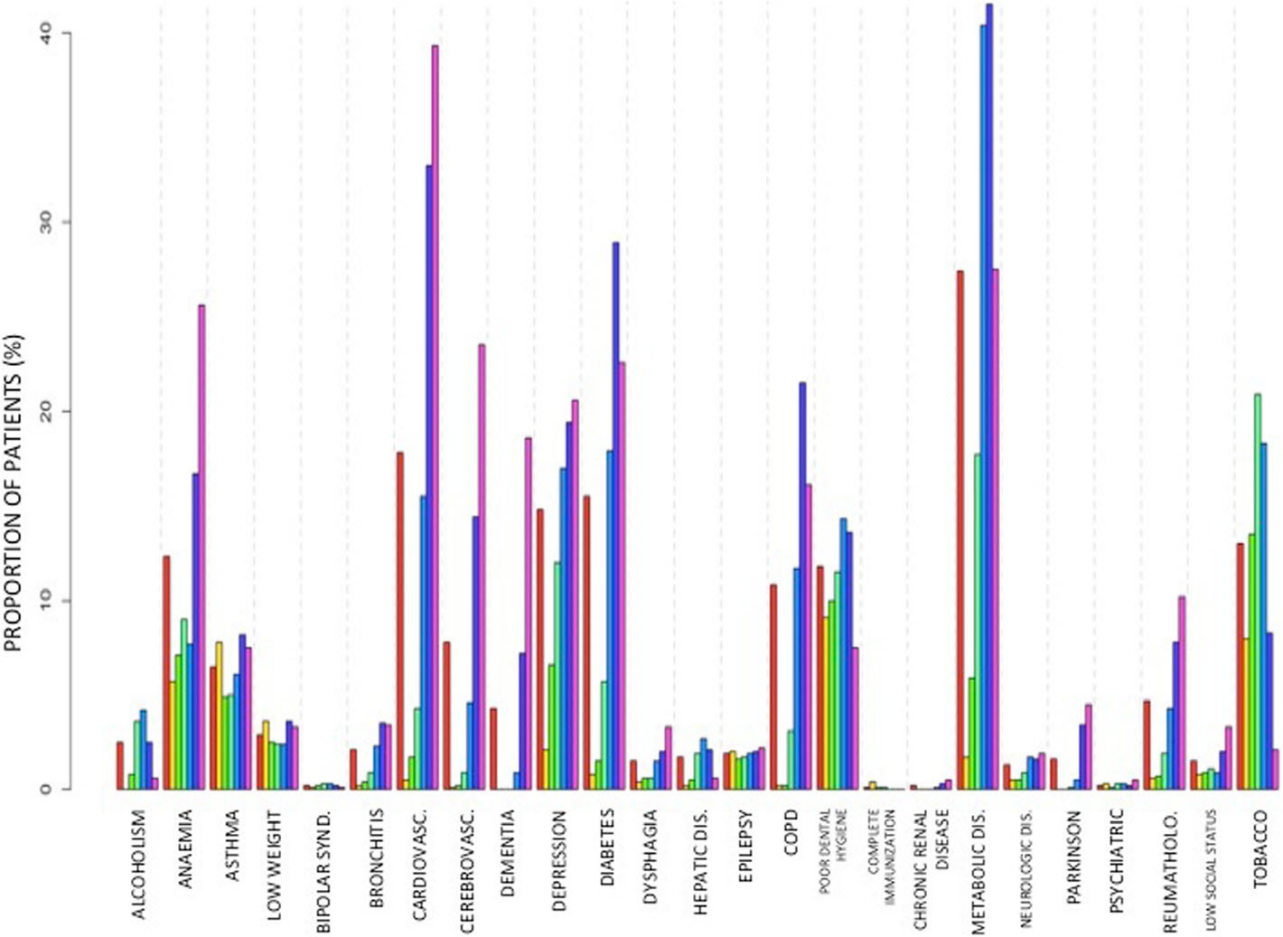
Distribution of the different lifestyle risk factors and comorbidities in patients seeking medical assistance because of pneumonia at primary care, according to age. Legend:

**Fig. 2:**
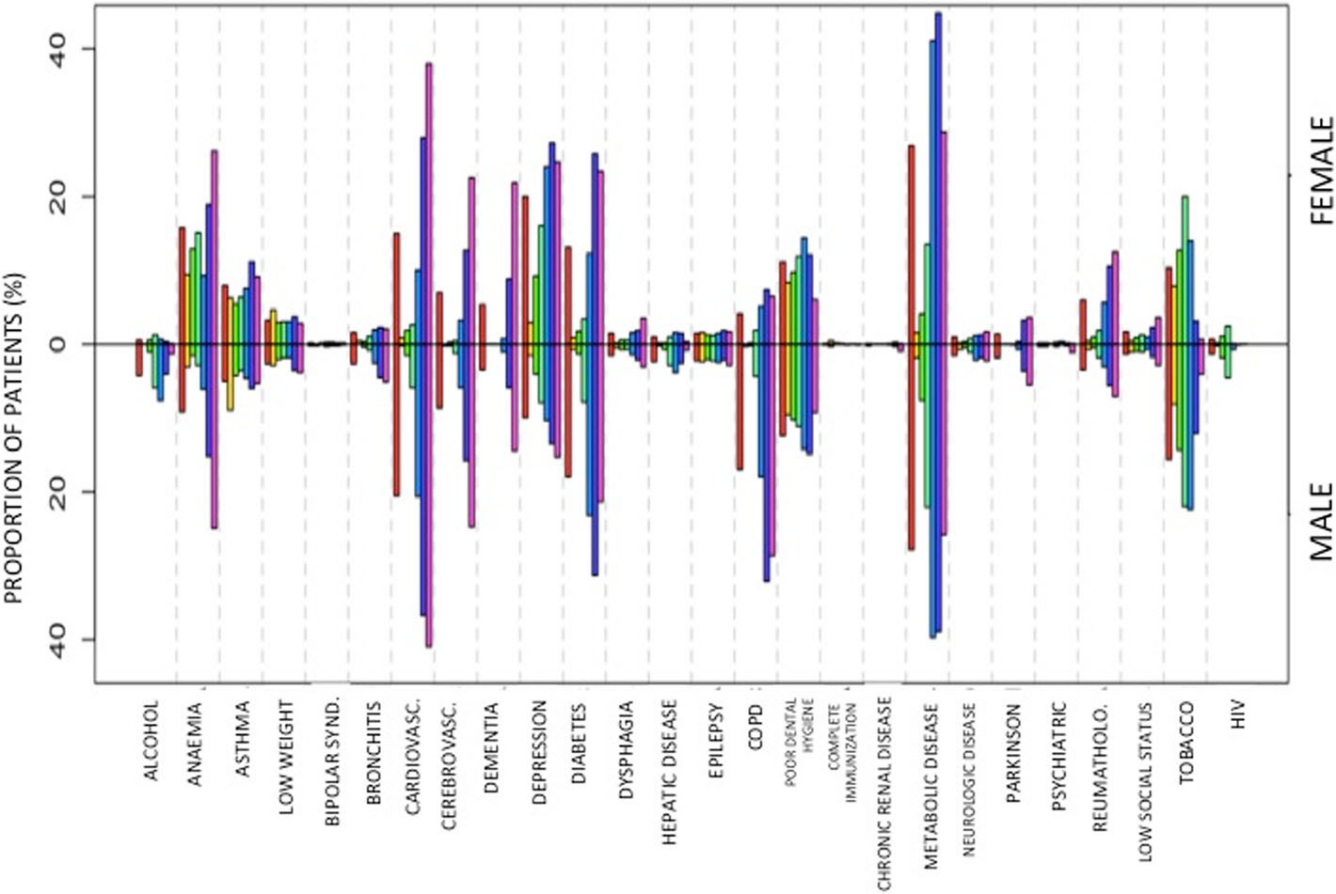
Distribution of the different lifestyle risk factors and comorbidities in patients seeking medical assistance because of pneumonia at primary care, according to gender. Legend:

Overall, the main risk factors detected were: metabolic disease (27.4 %), cardiovascular disease (17.8 %), diabetes (15.5 %), depression (14.8 %), smoking (13.0 %), anemia (12.3 %), poor dental hygiene (11.8 %), chronic obstructive pulmonary disease (COPD) (10.8 %) and cerebrovascular disease (7.8 %) (Figs. 1 and 2).

The main risk factors according to age were (Fig. 1): between 18 and 25 years: 9.1 % poor dental hygiene, 8.0 % smoking, 7.8 % asthma, 5.7 % anemia and 3.6 % low weight; between 25 and 40 years: smoking 13.5 %, 10.0 % poor dental hygiene, 7.1 % anemia, 6.6 % depression, 5.9 % metabolic disease and 4.9 % asthma; between 40 and 55 years: smoking 20.9 %, metabolic disease 17.7 %, depression 12.0 %, poor dental hygiene 11.5 %, and anemia 9 %; between 55 and 70 years: 40.4 % metabolic disease, smoking 18.3 %, 17.9 % diabetes, 17.0 % depression, 15.5 % cardiovascular disease, 14.3 %, poor dental hygiene and 11.7 % COPD; between 70 and 85 years: 41.5 % metabolic disease, 33.0 % cardiovascular disease, 28.9 % diabetes, COPD 21.5 %, 19.4 % depression, anemia 16.7 % and 14.4 % cerebrovascular disease; over 85 years: 39.3 % cardiovascular disease, 27.5 % metabolic disease, 25.6 % anemia, 23.5 % cerebrovascular disease, 22.6 % diabetes, 20.6 % depression, 18.6 % dementia and 16.1 % COPD.

**Fig. 3:**
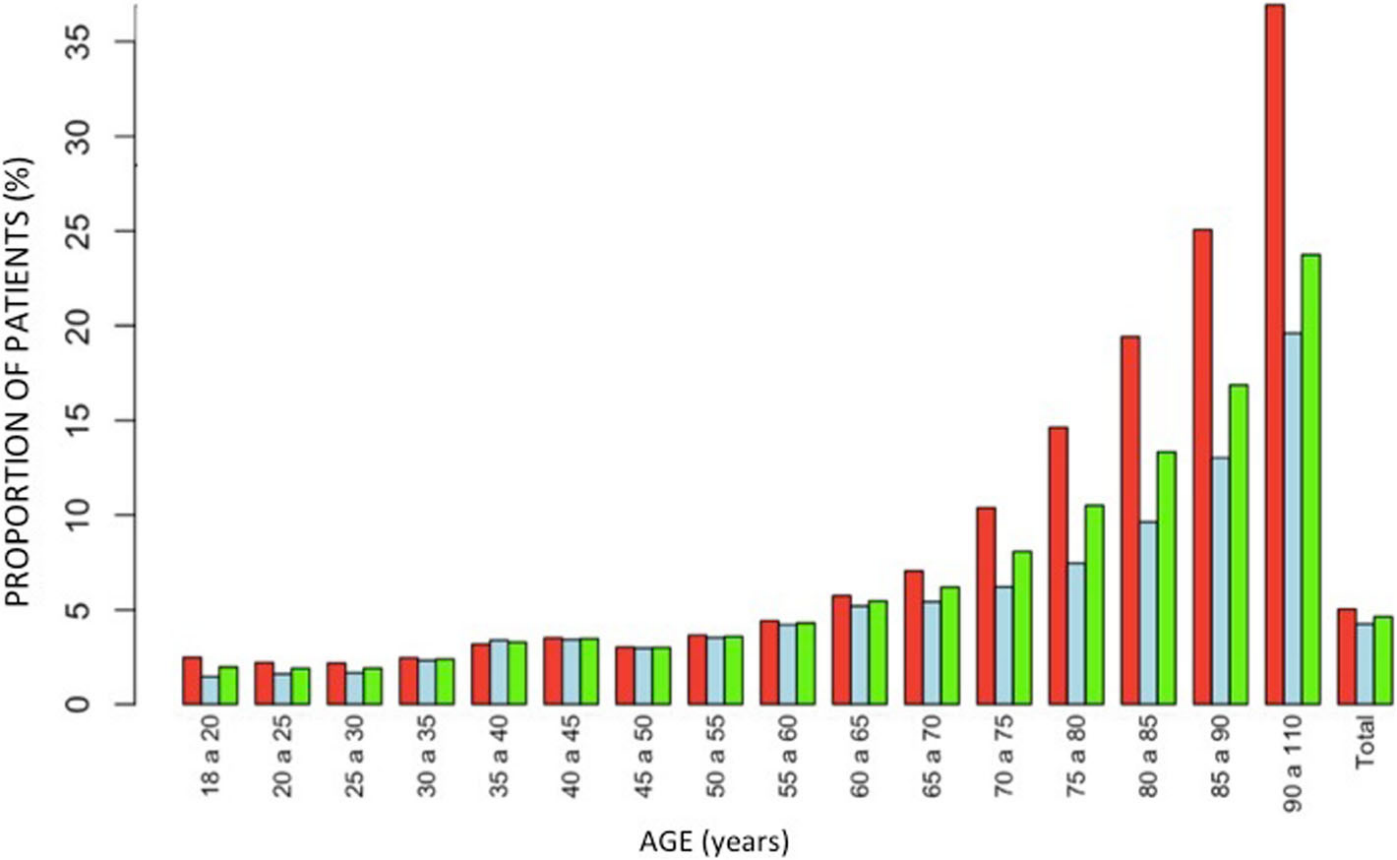
Global incidence of CAP stratified by age. Legend:

The main risk factors according to gender were (Fig. 2): women: metabolic disease (26.9 %), depression (20.0 %), anemia (15.8 %) and cardiovascular disease (15.0 %); men: metabolic disease (27.8 %), cardiovascular disease (20.5 %), diabetes (17.9 %) and smoking (15.5 %). Overall, taking into account the total number of risk factors and stratifying them by age, a progressive increase with age is observed, with an exponential increase in males, which even doubles the risk from 75 years of age (Fig. 3).

**Fig. 4:**
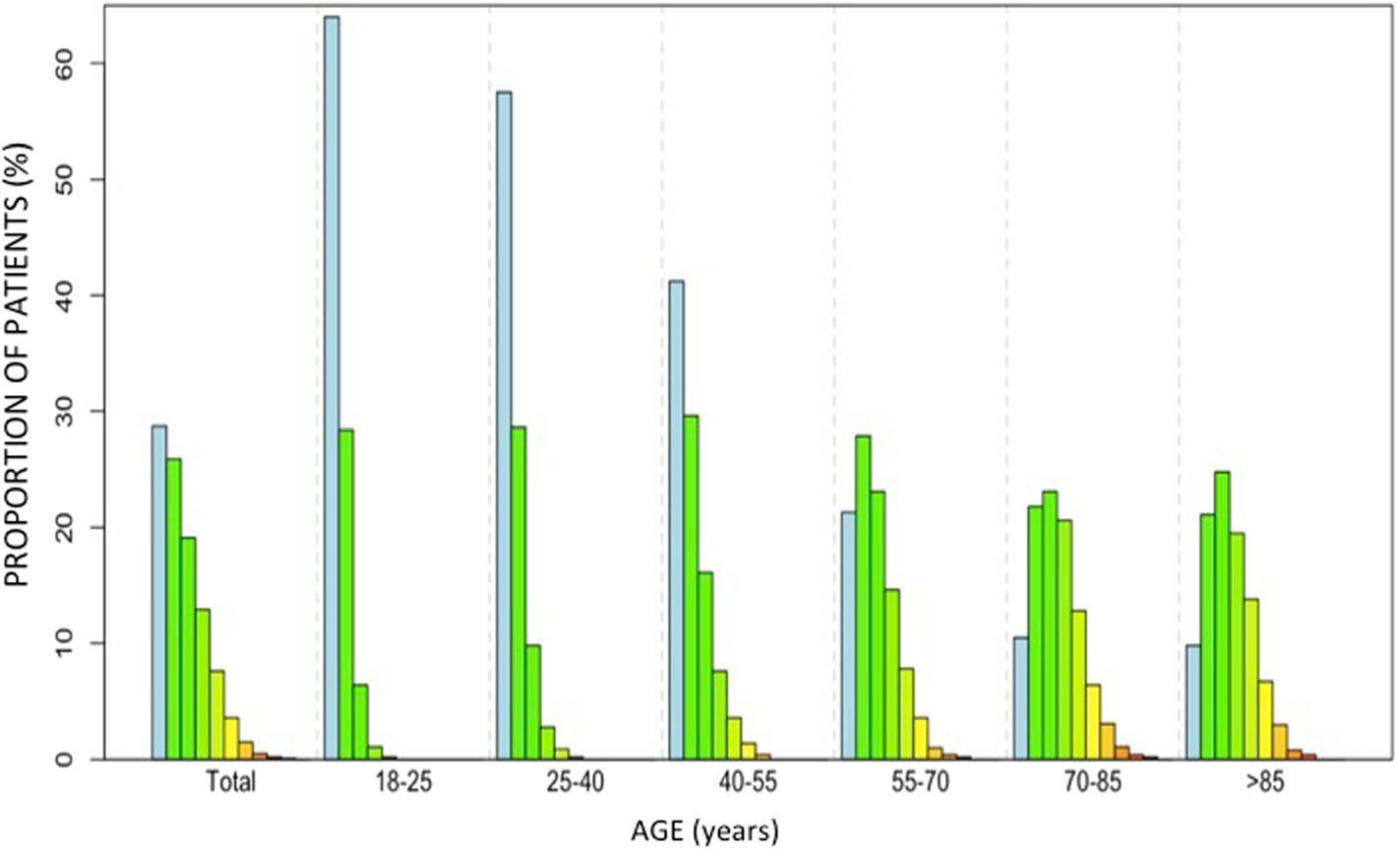
Global prevalence of the different lifestyle risk factors and comorbidities stratified by age. Legend:

Analyzing the number of comorbidities in terms of age, we note that less than 40 % of 18–25 year-old patients years showed any known risk factor, whereas after age 55 there is an identified risk factor in the 85.7 % of cases (one risk factor in 23.8 %, two factors in 23.4 %, three in 18.2 %, four in 11.2 %, five in 5.5 % and six in 2.3 %) (Fig. 4). The association of comorbidities showed no significant differences regarding sex in any age group.

## Discussion

Our findings, based on the largest primary care population database in Spain, indicate a high incidence of CAP in primary care adults in Spain that is strongly associated with a high prevalence of multiple lifestyle risk factors and comorbidities. If we do not implement appropriate interventions, the currently high and increasing prevalence of these associated risk factors will likely lead to an even higher incidence of CAP in the aging population of Spain.

The global incidence of CAP in adults calculated in this study (4.63/1000 person-years) is coherent with the estimates from other Spanish studies, which range from 1.6 to 9 episodes per 1000 person-years [7]. According to these results, the percentage of CAP is higher in Spain compared to other European countries. As suggested by other publications, this may be associated with intrinsic differences in the structure of the health system and other factors such as heterogeneity in the entry criteria, or the possibility of care in the emergency unit, among others [13]. Furthermore, our results confirm the previous observations of higher incidence rates associated with men and older ages [14–16].

We found the highest burden of disease associated with metabolic disease, cardiovascular disease and diabetes. These findings are consistent with pooled data from observational studies which demonstrated that patients with other medical conditions such as chronic respiratory diseases, cardiovascular diseases, cerebrovascular diseases, dementia and diabetes mellitus were the most frequently observed comorbidities associated with CAP [10, 11, 16]. Up to two-thirds of patients had a chronic respiratory disease and almost half had a chronic cardiovascular disease, highlighting the need for appropriate management of these patients to reduce their risk of CAP.

Lifestyle factors such as smoking, high alcohol intake or being underweight have been associated with an increased risk of CAP [10, 11, 16], which is consistent with our findings. Smoking and excessive alcohol consumption are major health risks globally and are targets for interventions to reduce the global burden of disease. Ensuring that patients make appropriate lifestyle changes would help reduce the overall burden of CAP. Similarly, being underweight may predispose patients to CAP due to the consequences of undernutrition or underlying conditions on immune function, so assessment of the nutritional status of vulnerable patients might help identify those at increased risk of CAP.

Overall, we observed substantially increased rates of pneumonia among persons with >1 risk condition, and disease rates were especially high among those with ≥3 risk conditions. This is especially significant when noting that risk factors tend to increase over time: after age 55, we identified a risk factor in 85.7 % of cases, and risk even doubles from age 75. This phenomenon of “risk stacking”— whereby risk of disease increases with increasing numbers of risk factors —has been noted for other diseases, for example, osteoporotic hip fractures and cardiovascular events [17, 18].

One potential limitation of the study is the underestimation of CAP incidence, as doubtful cases where there was not sufficient information in the medical history, or diagnosis of presumption was made without radiological confirmation, were excluded. Another aspect to bear in mind is that not all patients are initially diagnosed in primary care. It is not uncommon for a person to go directly to the hospital without going through the general practitioner, and such cases would not be initially recorded in our database. However, patients are commonly referred to their general practitioner for follow-up, or they inform the doctor about the episode suffered during further consultation in the health center. Thus, even though some cases may initially visit the emergency room or be hospitalized, they can be classified as CAP, provided that the doctor registers the episode in the patient’s medical history.

One of the strengths of BIFAP is that it is one of the largest primary care databases available. It includes information provided by 2692 family physicians and primary care pediatricians from the National Health System, integrating information from 4,800,207 valid anonymized medical records for a total of 24,957,871 person-years of follow-up (5-year average patient tracking); including: 76,561,939 records of health problems, 414,852,056 medication records, 14,190,861 immunization records and 674,846,412 general records of patient data.

The organization of the Spanish public health system sets the primary care as the main gateway to other health services. Thus BIFAP includes a wealth of information on when, how and why patients visit primary care centers. Although the objective behind the development of this database was to facilitate pharmacoepidemiological studies, its potential is greater. With appropriate methods and after overcoming complex validation tests, BIFAP data can be used to calculate incidence and prevalence of diseases from thousands of patients, and to investigate risk factors and associated prognostic factors [19, 20]. This research is a population-based study, which could determine accurately the incidence and prevalence of risk factors of CAP in Spain, and demonstrates the utility of BIFAP as a resource to estimate the prevalence and incidence of disease.

## Conclusions

The incidence of CAP in primary care adults in Spain is high, increases with age, and it is double in males as compared to females over 75 years of age. The main risk factors associated with CAP are metabolic disease, cardiovascular disease, and diabetes, all of them increasing nowadays, which could favor further increase in CAP incidence. Additional studies assessing risk stratification and a better understanding of the individual phenotypes with the greatest risk of CAP could help the development and implementation of the appropriate interventions in order to reduce the risk of infection and burden of CAP at the earliest level of medical care.

## Additional file

Additional file 1: Different lifestyle risk factors and comorbidities associated to the first register of pneumonia. (DOCX 42 kb)

## Abbreviations

AEPAP: Asociación Española de Pediatría de Atención Primaria
BIFAP: Computerized Database for Pharmacoepidemiological Studies in Primary Care
CAP: Community-acquired pneumonia
SEMERGEN: Sociedad Española de Médicos de Atención Primaria
SEMFYC: Sociedad Española de Medicina Familiar y Comunitaria
