# Erratum to: Incidence and risk factor prevalence of community-acquired pneumonia in adults in primary care in Spain (NEUMO-ES-RISK project)

**DOI:** 10.1186/s12879-016-2134-6

**Published:** 2017-01-12

**Authors:** I. Rivero-Calle, J. Pardo-Seco, P. Aldaz, D. A. Vargas, E. Mascarós, E. Redondo, J. L. Díaz-Maroto, M. Linares-Rufo, M. J. Fierro-Alacio, A. Gil, J. Molina, D. Ocaña, Federico Martinón-Torres, on behalf of NEUMOEXPERTOS group

**Affiliations:** 1 Translational Pediatrics and Infectious Diseases Section, Pediatrics Department, Hospital Clínico Universitario de Santiago de Compostela, Travesía da Choupana, s/n, 15706 Santiago de Compostela, Spain; 2 Genetics, Vaccines, Infections and Pediatrics Research Group (GENVIP), Healthcare Research Institute of Santiago de Compostela, Santiago de Compostela, Spain; 3 Member of the Infectious Diseases Prevention Group PAPPS-SEMFYC, Primary Health Care Center San Juan, Pamplona, Spain; 4 Versatile Hospitalization Unit, Hospital de Alta Resolución El Toyo, Agencia Pública Sanitaria, Hospital de Poniente, Almería, Spain; 5 Health Department, Hospital Dr Peset, Primary Care Center Fuente de San Luís, Valencia, Spain; 6 Preventive and Public Health Activities Group SEMERGEN, International Heath Center, Madrid, Spain; 7 Primary Care Health Center Guadalajara, Infectious Diseases Group SEMERGEN, Guadalajara, Spain; 8 Primary Care and Clinical Microbiology, Infectious Diseases Group SEMERGEN, Fundación io, Spain; 9 Primary Care Health Center El Olivillo, Cádiz, Spain; 10 Preventive Medicine and Public Health, Rey Juan Carlos University, Madrid, Spain; 11 Primary Care Respiratory Group, Health Care Center Francia, Fuenlabrada, Madrid Spain; 12 Primary Care Respiratory Group, Health Care Center Algeciras, Algeciras, Spain

## Erratum

In the original publication of this article [1], the figure files provided were incorrect. The correct figures can be found at the end of this text.

**Fig. 1:**
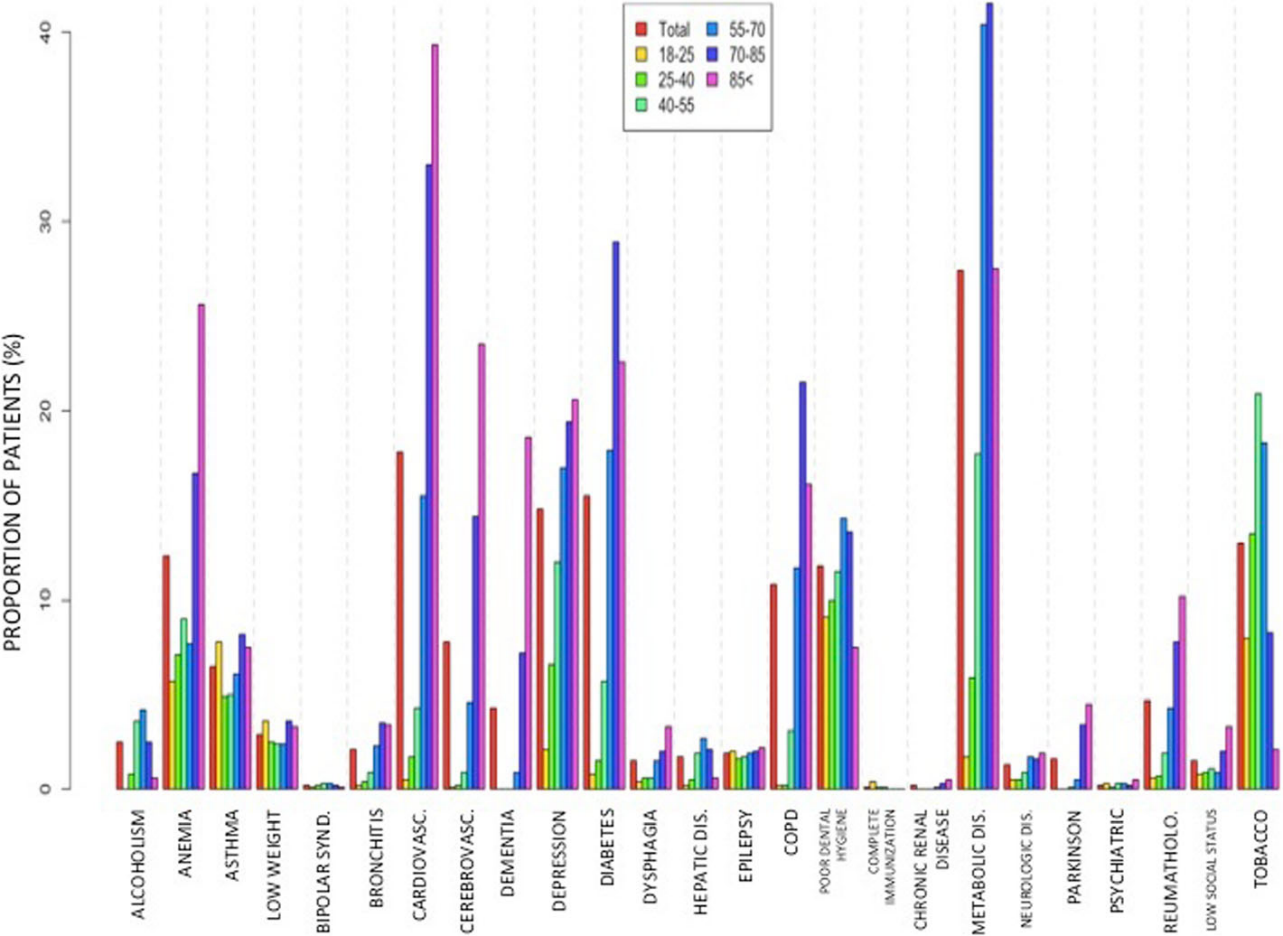
Distribution of the different lifestyle risk factors and comorbidities in patients seeking medical assistance because of pneumonia at primary care, according to age

**Fig. 2:**
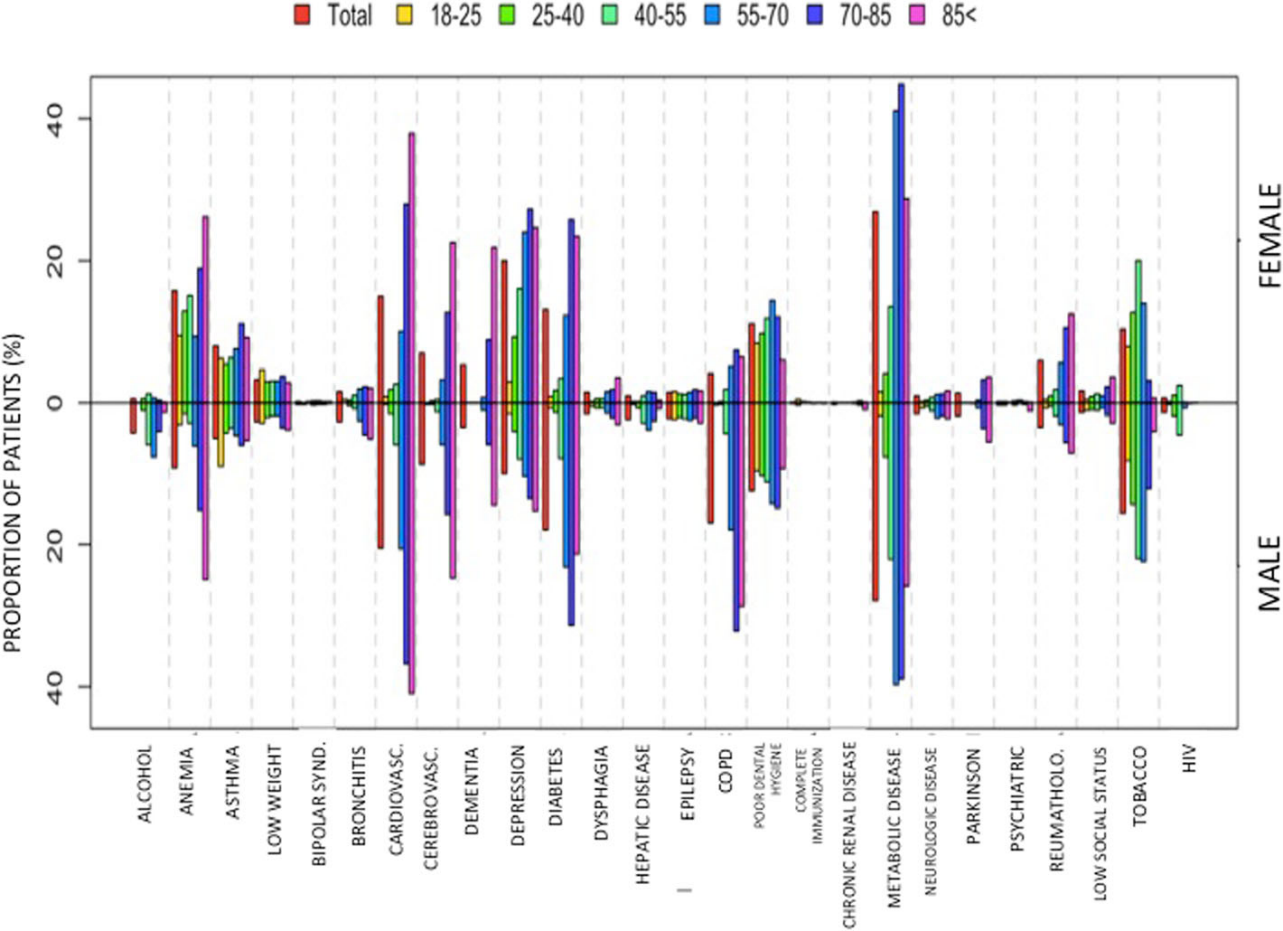
Distribution of the different lifestyle risk factors and comorbidities in patients seeking medical assistance because of pneumonia at primary care, according to gender

**Fig. 3:**
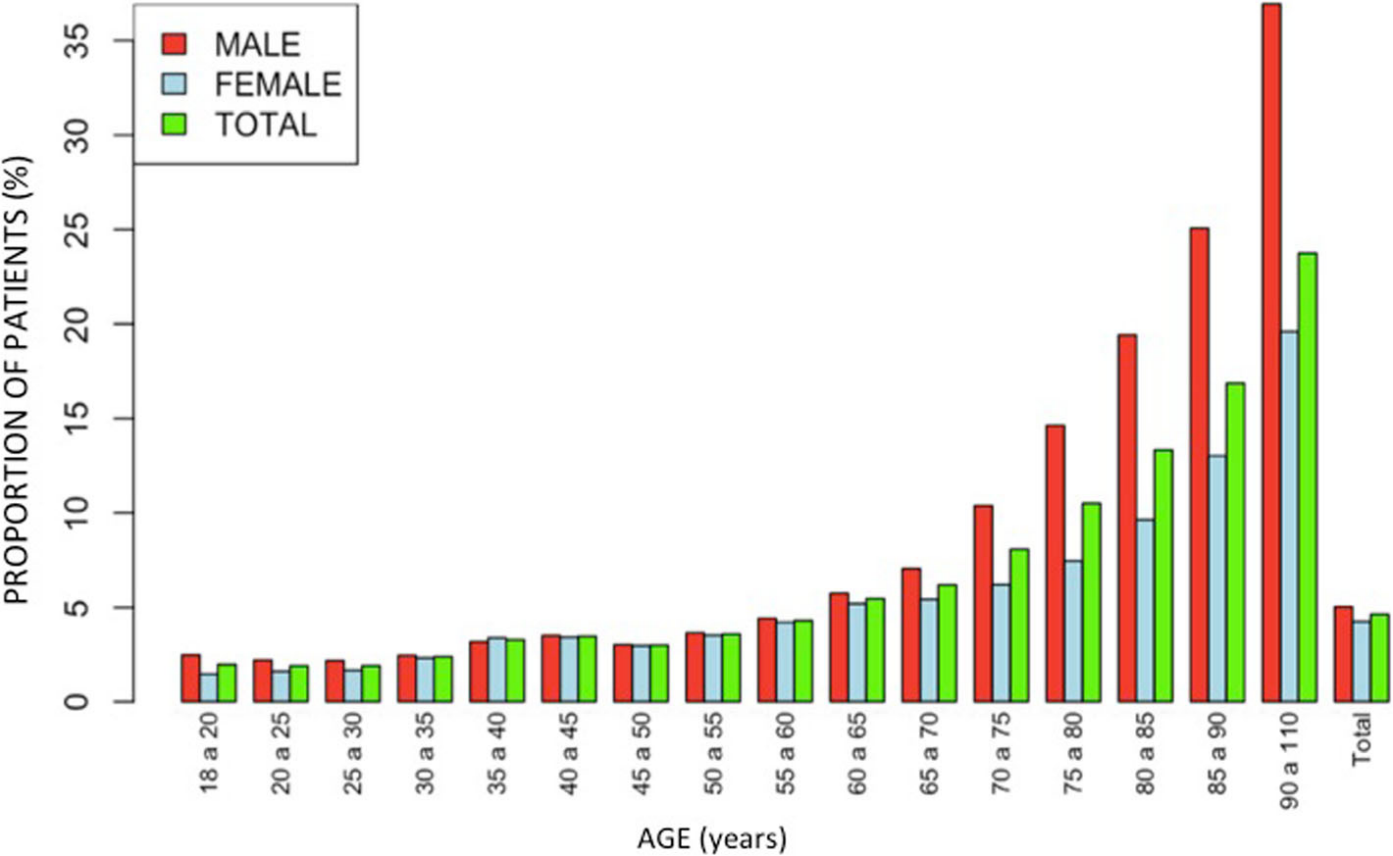
Global incidence of CAP stratified by age

**Fig. 4:**
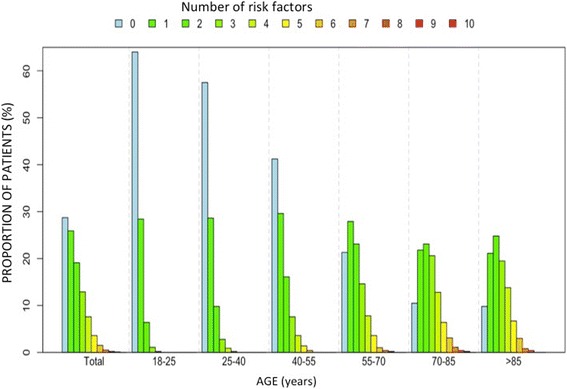
Global prevalence of the different lifestyle risk factors and comorbidities stratified by age

Furthermore, the acknowledgments section omitted the following information in the original article [1]:

"The data for the realization of this publication are part of the database BIFAP managed by the Spanish Agency of Medicines and Sanitary Products (AEMPS). The results, discussion and conclusions of this study are those perceived by the authors only and do not represent in any way the position of the AEMPS on this subject.

The authors are grateful for the excellent collaboration of family physicians and primary care pediatricians who participated in BIFAP".
