# Supplementary material for: Incidence and risk factor prevalence of community-acquired pneumonia in adults in primary care in Spain (NEUMO-ES-RISK project)
Source: BMC Infect Dis. 2016 Nov 7;16:645. doi: 10.1186/s12879-016-1974-4 (PMC5100110; doi:10.1186/s12879-016-1974-4)
Supplement: Additional file 1: — Different lifestyle risk factors and comorbidities associated to the first register of pneumonia. (DOCX 42 kb) [file 12879_2016_1974_MOESM1_ESM.docx]

**Additional file 1**

| **RISK FACTOR** | **CIE-9** | **CIAP-2** |
| --- | --- | --- |
| **Low socio-economic status** |  | Z01, Z02, Z04, Z05, Z08 |
| **Smoking** |  | P17.01, P17.02, P17.03, P17.04, P17.05, P17.07 |
| **Alcohol abuse** |  | P15.02, P15.03, P15.04, P15.05, P15.06, P15.07, P15.10, P15.16, P15.18 |
| **Nutritional status.** Underweight |  | T91.03, T91.04, T8.02, T8.04, T29.15 |
| **Poor dental hygiene** |  | D82.01, D82.02, D82.04, D82.05, D82.06, D82.07, D82.08, D82.08, D82.14, D82.15, D82.29, D82.30 |
| **Cardiovascular disease**   - Chronic heart disease - Heart failure - Atherosclerosi**s** | 410-414, 96.0, V45.81, 45.82, 398.91, 402.01, 402.11, 402.91, 404.01, 404.03, 404.11, 404.13, 404.93, 428.0, 428.2, 428.3, 428.4 | K22.01, K22.02, K22.03, K22.04, K74.01, K74.03, K74.04, K74.05, K74.06, K74.07, K74.08, K74.09, K74.10, K74.11, K74.12, K74.14, K74.15, K74.16, K74.17, K75.01, K75.02, K75.03, K75.04, K75.06, K75.07, K75.08, K75.09, K76.01, K76.02, K76.03, K76.04, K76.05, K76.06, K76.07, K76.08, K76.09, K76.10, K76.11, K77.02, K77.03, K77.04, K77.05, K77.07, K77.08, K77.09, K77.11 , K84.24, |
| **Cerebrovascular disease**  **-** Stroke | 094.87, 346.6, 430.0-438.9, 747.81, 784.3, 997.02 | K89, K90, K91 |
| **CNS disease**   - Parkinson’s disease - Epilepsy - Multiple sclerosis - Neurological disorders |  | N87.02, N88.03, N88.04, N88.05, N88.06, N88.07, N99.04, N99.05, N99.16, N99.17 |
| **Psychiatric conditions**   - Depression - Bipolar disorder - Dementia | 295.0-295.90, 296.20, 296.30, 296.50, 296.82, 296.90, 298.0, 300.4, 309.0, 309.1, 311,  296.40-296.60, 290.0-290.20, 290.40, 294.10, 331.0, 331.1, 331.11, 331.19, 331.2 | P76.01, P76.02, P76.03, P76.04, P76.05, P76.06, P76.07, P76.08, P76.10, P76.11, P76.12, P76.14, P76.15, P76.16, P20.07, P20.08 |
| **Dysphagia** |  | D21.02, D21.03 |
| **Anemia** |  | B80.01, B80.05, B80.07, B81.02, B81.03, B81.04, B81.05, B81.06, B81.07, B82.02, B82.05, B82.06, B82.07, B82.08, B82.08, B82.09, B82.10 |
| **Chronic respiratory disease**   - COPD - Bronchitis - Asthma - History of respiratory tract infection in the past year | 491.0, 491.2, 491.8, 491.9, 492, 494.0, 496, 493.0, 493.1, 493.2, 493.9 | R96.19, R96.20, R96.21, R96.22, R99.13, R99.14, R96.16 |
| **Chronic renal disease** | 403.01-403.93, 582.0-588.9 | U99.16, U99.17, U99.18, U99.19 |
| **Chronic liver disease. Cirrhosis** |  | D97.04, D97.06, D97.07, D97.07, D97.09, D97.11, D97.16, D97.17, D97.18 |
| **Diabetes mellitus** | 250.01-03, 250.10-13, 250.20-23, 250.30-33, 250.40-43, 250.50-53, 250.60-63, 250.70-73, 250.80-83, 250.90-93 | T89.01, T89.02, T89.04, T89.05, T89.06, T90.02, T90.03, T90.04, T90.08, T90.09, T90.10, T90.13, T90.14, T90.15 |
| **Arthrosis**   - Rheumatoid arthritis | 715.0-715.30, 715.80, 715.90, 720.1, 720.2, 720.8, 720.81, 720.89, 720.9, 721.0, 721.90, 722.0, 722.10, 722.30, 722.70, 722.80, 722.90, 723.0, 724.0, 724.70 | L88.01, L88.02, L88.07, L88.08, L91.15, L91.16, L91.17, L91.19, L91.22, L91.23, L91.24, L91.25, L91.26, L91.28, L91.29 |
| **Metabolic disease**   - DislipemIa - Hipercolesterolemia - Hipertrigliceridemia |  | T93.01, T93.02, T93.03, T93.04, T93.05, T93.06, T93.07, T93.08, T93.09, T93.10, T93.11, T93.12, T93.13, T93.14, T93.15, T93.16, T93.19, T93.20, T99.14 |
| **HIV infection** | 42, 079.53, V08 | B90.01, B90.02, B90.03, B90.04, B90.05 |
| **Pneumococcal Vaccination** |  | A44.15 |
